# The Downregulation of ADAM17 Exerts Protective Effects against Cardiac Fibrosis by Regulating Endoplasmic Reticulum Stress and Mitophagy

**DOI:** 10.1155/2021/5572088

**Published:** 2021-05-06

**Authors:** Chang Guan, Hai-Feng Zhang, Ya-Jing Wang, Zhi-Teng Chen, Bing-Qing Deng, Qiong Qiu, Si-Xu Chen, Mao-Xiong Wu, Yang-Xin Chen, Jing-Feng Wang

**Affiliations:** ^1^Department of Cardiology, Sun Yat-sen Memorial Hospital, Sun Yat-sen University, Guangzhou, China; ^2^Laboratory of Cardiac Electrophysiology and Arrhythmia in Guangdong Province, Guangzhou, China; ^3^Department of Otolaryngology, Sun Yat-sen Memorial Hospital, Sun Yat-sen University, Guangzhou, China

## Abstract

**Background:**

A disintegrin and metalloproteinase 17 (ADAM17) is a transmembrane protein that is widely expressed in various tissues; it mediates the shedding of many membrane-bound molecules, involving cell-cell and cell-matrix interactions. We investigated the role of ADAM17 within mouse cardiac fibroblasts (mCFs) in heart fibrosis.

**Methods:**

mCFs were isolated from the hearts of neonatal mice. Effects of ADAM17 on the differentiation of mCFs towards myofibroblasts and their fibrotic behaviors following induction with TGF-*β*1 were examined. The expression levels of fibrotic proteins, such as collagen I and *α*-SMA, were assessed by qRT-PCR analysis and western blotting. Cell proliferation and migration were measured using the CCK-8 and wound healing assay. To identify the target gene for ADAM17, the protein levels of the components of endoplasmic reticulum (ER) stress and the PINK1/Parkin pathway were assessed following ADAM17 silencing. The effects of ADAM17 silencing or treatment with thapsigargin, a key stimulator of acute ER stress, on mCFs proliferation, migration, and collagen secretion were also examined. *In vivo*, we used a mouse model of cardiac fibrosis established by left anterior descending artery ligation; the mice were administered oral gavage with a selective ADAM17 inhibitor (TMI-005) for 4 weeks after the operation.

**Results:**

We found that the ADAM17 expression levels were higher in fibrosis heart tissues and TGF-*β*1-treated mCFs. The ADAM17-specific siRNAs decreased TGF-*β*1-induced increase in the collagen secretion, proliferation, and migration of mCFs. Knockdown of ADAM17 reduces the activation of mCFs by inhibiting the ATF6 branch of ER stress and further activating mitophagy. Moreover, decreased ADAM17 expression also ameliorated cardiac fibrosis and improved heart function.

**Conclusions:**

This study highlights that mCF ADAM17 expression plays a key role in cardiac fibrosis by regulating ER stress and mitophagy, thereby limiting fibrosis and improving heart function. Therefore, ADAM17 downregulation, within the physiological range, could exert protective effects against cardiac fibrosis.

## 1. Introduction

Cardiac fibrosis is a common pathophysiological process that exists in various heart diseases such as myocardial infarction, hypertension, and cardiac hypertrophy, and it is characterized by excessive deposition of extracellular matrix (ECM) proteins [1, 2]. This process is a strategy to protect heart function in the early stage, and it can repair, replace, and strengthen the severely or chronically damaged tissue. However, long-term excessive deposition of ECM will reduce the compliance of the ventricle and the contractile function of cardiomyocytes, eventually leading to heart failure and increasing mortality [3, 4]. It is an inappropriate transformation process of quiescent cardiac fibroblasts (CFs) to myofibroblasts (CMFs), leading to the disproportionate accumulation of ECM proteins [4–6]. Due to the clear association between cardiac fibrosis and its adverse outcomes, understanding the mechanisms responsible for the pathogenesis of cardiac fibrosis and formulating new antifibrotic strategies for treating patients with heart diseases are issues requiring urgent attention.

A disintegrin and metalloproteases (ADAMs) is a newly discovered protein family in recent years. Numerous members of this family have been reported to be involved in the regulation of cardiovascular diseases [7, 8]. A disintegrin and metalloproteinase 17 (ADAM17), as one of the most well-studied proteins in the ADAMs family, is widely expressed in various tissues including the heart, kidney, liver, and lung. As a transmembrane protein, its main function is to cleave extracellular domains of various substrate proteins, such as TNF*α*, TNF receptors (TNFR1 and TNFR2), and several epidermal growth factor receptor (EGFR) ligands [9, 10]. The cells also contain a large amount of ADAM17, but the mechanism of its action remains unclear [11]. It plays an important role in biologic processes involving cell-cell and cell-matrix interactions. Many studies have shown that ADAM17 can participate in the regulation of the occurrence and development of tumors, inflammatory diseases, nervous system, and cardiovascular diseases. In the cardiovascular system, a short-term increase in ADAM17 expression after acute myocardial infarction can aggravate cardiac dysfunction [12]. The deficiency of ADAM17 in vascular smooth muscle cells can inhibit the progression of thoracic aortic aneurysms [13]. ADAM17 in blood vessels can aggravate angiotensin II-induced vascular remodeling and peritubular fibrosis [14, 15]. In fibrotic diseases, ADAM17 and connective tissue growth factor (CTGF) participate in the formation of pulmonary fibrosis [16]. The expression of ADAM17 is increased in glomerular sclerosis and interstitial fibrosis and correlated positively with the progression of renal fibrosis [17–19]. However, whether ADAM17 plays functional roles during the development of cardiac fibrosis is still unknown.

As important intracellular organelles, the endoplasmic reticulum (ER) and mitochondria are widely involved in the regulation of protein, lipid, and energy metabolism and maintain the normal function of cells. Many studies have shown that there is a physical and functional interaction between the ER and mitochondria. This interaction is involved in the regulation of atherosclerosis, myocardial hypertrophy, heart failure, and other cardiovascular diseases [20]. ER stress is a condition that is accelerated by the accumulation of unfolded/misfolded proteins after disturbances owing to a variety of physiological and pathological phenomena [21]. Mitophagy, i.e., selective mitochondrial degradation through autophagy, is a conserved cellular process used to eliminate damaged mitochondria. Under various pathological stimuli, ER stress and mitophagy are activated. ER stress leads to increased synthesis of molecular chaperones (such as GRP78) through three pathways (IRE1/XBP1s, PERK/ATF4, and ATF6), decreased protein synthesis, and increased degradation. Mitophagy degrades impaired mitochondria through the classic PINK1/Parkin pathway [22]. Excessive ER stress will aggravate abnormal Ca^2+^ and ROS regulation and further damage mitochondria, aggravate mitophagy, and eventually lead to cell death [21–23]. ER stress and mitophagy likely contribute to the development and progression of cardiovascular diseases such as heart failure and atherosclerosis [24–26]. Furthermore, excessive ER stress and the absence of mitophagy both lead to the aggravation of cardiac fibrosis, and both of them are key mechanisms in cardiac fibrosis [27–30]. Recent studies have shown that ADAM17 can affect the progression of vascular remodeling, perivascular fibrosis, and differentiation and drug resistance of cancer cells through ER stress and mitophagy [14, 31]. However, the role of ADAM17, ER stress, and mitophagy in cardiac fibrosis remains unclear.

In this research, we speculated the potential effects of ADAM17 on cardiac fibrosis. To address this question, the present study examined the function of ADAM17 with ER stress and mitophagy in cardiac fibrosis. We found that reduction of ADAM17 expression is associated with beneficial effects against activation of mCFs by regulating the ATF6 branch of ER stress and the PINK1/Parkin pathway of mitophagy. Furthermore, inhibition of ADAM17 in mice could ameliorate left anterior descending artery (LAD) ligation-induced cardiac fibrosis and improve heart function.

## 2. Methods

### 2.1. Induction of Myocardial Infarction (MI) and Treatment

All animal care and experimental protocols were performed in accordance with the Institutional Animal Care and were approved by the Ethics Committee of the Sun Yat-sen University. Male C57BL/6 mice (6–8 weeks old, 20–25 g) were subjected to the induction of MI by LAD ligation, as described previously [32]. Briefly, the mice were anesthetized with sodium pentobarbital (intraperitoneal injection, 50 mg/kg; Merck) and mechanically ventilated using the HX-101E ventilator (Chengdu Tai Meng Software Ltd.). Their chests were opened through the 4th left intercostal space, and then, the LAD was ligated with 8-0 polyester sutures. Mice in the sham group were subjected to the same experimental procedures but without LAD constriction. The mice were randomly divided into four groups (*n* = 6 per group): control/MI groups receiving vehicle buffer (2% Tween 80 and 0.5% methylcellulose) by oral gavage twice daily and TMI-005/MI+TMI-005 groups receiving 10 mg/kg ADAM17 inhibitor (TMI-005, Glpbio, GC35377) by oral gavage twice daily. After 4 weeks of intragastric administration (endpoint), cardiac function was assessed by echocardiography analysis, and then, the hearts were harvested.

### 2.2. Isolation and Culture of CFs

First, mouse cardiac fibroblasts (mCFs) were isolated and cultured as described in a previous study [33]. mCFs were isolated from 1- to 3-day-old neonatal mice obtained from the Animal Centre of the Sun Yat-sen University. Briefly, the hearts of mice were quickly excised and digested with 0.1% trypsin/D-Hanks solution at 4°C with gentle rotation for 12 hours. Then, the hearts were digested with 0.8 mg/mL collagenase II/D-Hanks solution at 37°C for 10 minutes and were repeated 2–3 times. Cells were collected and suspended in Dulbecco's modified Eagle medium: Nutrient Mixture F12 (DMEM/F12, Gibco, Thermo Fisher Scientific) containing 10% fetal bovine serum. After plating at 37°C for 0.5 h, mCFs adhered onto the culture plates, and the nonadherent cells in the supernatant were removed by washing with PBS. Then, the mCFs were cultured with DMEM/F12 containing 10% FBS (Invitrogen, Carlsbad) and 1% penicillin/streptomycin (Gibco, Thermo Fisher Scientific) at 37°C in humid air containing 5% CO_2_, until they reached confluence; then, they were passaged further. The identity of mCFs was confirmed by immunofluorescence staining with vimentin. mCFs at the second or third passages were used in the subsequent experiments. After starvation in the serum-free medium for 12 h, the mCFs were treated with 5 ng/mL recombinant mouse TGF-*β*1 protein (R&D Systems, #7666-MB) for 48 h.

### 2.3. Transfection Procedure and TGF-*β*1 Administration

ADAM17-specific siRNAs were purchased from RiboBio (Guangzhou, China). mCFs were seeded on 6-well plates at 50–60% confluence before transfection. Each siADAM17 (50 nM) (Table s1) and Lipofectamine® RNAiMAX (Invitrogen) were mixed, incubated at room temperature for 10–15 min, and then added to the cell cultures. Twenty-four hours after transfection, the mCFs were treated with TGF-*β*1 (5 ng/mL) for 48 h or thapsigargin (TG, 1 *μ*M, Sigma-Aldrich, T9033) for 24 h.

### 2.4. Wound Healing Assay

To verify the migration ability of the mCFs, a wound healing assay was performed. Transfected mCFs (2 × 10^5^ cells/well) left untreated or those treated with TGF-*β*1 or thapsigargin were seeded into 6-well plates and grown until subconfluence. A scratch was then created in each well using a 200 *μ*L pipette tip, and the wounded monolayers were washed twice with PBS to remove the cell debris and floating cells. The wounds were photographed at the beginning (time 0 h) and then after 24 h under an inverted microscope, and the width of the wound area was measured at the reference points ImageJ software.

### 2.5. CCK-8 Assay

The CCK-8 method was used to analyze the proliferation of mCFs according to the manufacturer's instructions. The mCFs, transfected with siADAM17 as described above, were seeded into 96-well plates at a density of 5 × 10^3^ cells/well and treated with TGF-*β*1 or thapsigargin for a total of 24, 48, and 72 hours, respectively. Then, 100 *μ*L of fresh medium containing 10 *μ*L CCK-8 (DOJINDO) was added into the wells, followed by incubation in the presence of 5% CO_2_ at 37°C for 2 h; next, the absorbance readings of the samples were obtained at 450 nm using a microplate reader (Invitrogen).

### 2.6. Quantitative Real-Time RT-PCR

Total RNA was extracted from the mouse heart tissues or mCFs using the Trizol reagent (Invitrogen). A PrimeScript™ RT Master Mix Kit (Takara Bio, RR036A) was used to reverse-transcribe 1 *μ*g of RNA into cDNA, as described previously [4]. Quantitative real-time polymerase chain reaction (qRT-PCR) was performed using the SYBR method (Takara Bio, RR420A) in a LightCycler® 96 Real-Time PCR System (Roche). The qRT-PCR analysis was performed using the primers listed in Table s2. Normalization of gene expression was achieved by comparing the expression of the target genes to that GAPDH in the corresponding samples.

### 2.7. Western Blotting

Total proteins were extracted from the mCFs or mouse heart tissues using a radio immunoprecipitation assay (RIPA) buffer (CST) containing protease and phosphatase inhibitors (Roche, 04906845001, 04693124001). The mitochondrial and cytosolic fractions were isolated from cells using a commercially available kit (Thermo Fisher Scientific, #89874) according to the manufacturer's instructions. Total protein or mitochondrial protein concentrations were measured using a BCA Protein Assay Kit (Pierce, Thermo Fisher Scientific, 23227). Equal amounts of the total protein samples were separated by 10% sodium dodecyl sulfate-polyacrylamide gel electrophoresis; the resultant bands were transferred to 0.2 *μ*m PVDF membranes (Millipore). Then, the membranes were blocked with 5% BSA diluted with tris-buffered saline tween-20 at room temperature for 1 h and incubated overnight at 4°C with the following primary antibodies: antibodies against collagen I (1 : 1000, Abcam), *α*-SMA (1 : 1000, Abcam), ADAM17 (1 : 1000, Abcam), Smad2/3 (1 : 1000, CST), p-Smad2/3 (1 : 1000, CST), p-PERK (1 : 1000, CST), ATF4 (1 : 1000, Abcam), p-IRE1 (1 : 1000, Abcam), XBP1s (1 : 1000, Abcam), ATF6 (1 : 1000, Proteintech), GRP78 (1 : 1000, Abcam), and GAPDH (1 : 1000, CST). They were then washed with TBST (×3) and incubated with horseradish peroxidase-conjugated secondary antibody (1 : 5000, CST) for 1 h at room temperature. Next, the membranes were washed with TBST (×3), followed by the detection of the protein bands using ECL reagents (Merck Millipore, WBULS050). The intensity of the protein bands was quantified using the ImageJ software and normalized to the intensity of GAPDH.

### 2.8. ADAM17 Enzymatic Activity Assay

The ADAM17 activity assay was performed using the InnoZyme TACE Activity Kit (Sigma-Aldrich, CBA042) following the manufacturer's protocol. The activity of ADAM17 was measured using an internally quenched fluorescent substrate, MCA-KPLGL-Dpa-AR-NH2. The resultant fluorescence was measured at an excitation wavelength of 320 nm and an emission wavelength of 405 nm. The ADAM17 activity was expressed as relative fluorescence units per milligrams of protein.

### 2.9. Echocardiography

Echocardiography was performed to assess the left ventricular end systolic diameter (LVESD), left ventricular end diastolic diameter (LVEDD), left ventricular ejection fraction (LVEF), and left ventricular fractional shortening (LVFS) using a VisualSonics Echo System (Vevo 2100, VisualSonics, Inc.) equipped with a MicroScan Transduce (MS-400, 30 MHz, VisualSonics, Inc.). The mice were slightly anesthetized via inhalation of 1% isoflurane, and their heart rate was maintained at >400 bpm. The hearts' short axis views were obtained in B-mode and M-mode. The LVESD, LVEDD, LVEF, and LVFS were calculated from the M-mode measurements; the measurements represented the mean of three successive cardiac cycles, as described previously [34].

### 2.10. Masson's Trichrome Staining

The mouse hearts were dissected and fixed in 4% paraformaldehyde for 24 h; then, they were embedded in paraffin and stained with Masson's trichrome stain, as previously described [3]. The area occupied by collagen was calculated using the ImageJ software. The percentage of fibrosis was measured using the following formula: (fibrotic areas/total left ventricular areas) × 100%.

### 2.11. Statistical Analysis

All data are presented as the mean ± SEM. Statistical analysis was performed using Student's *t*-test for the comparison of two groups and one-way ANOVA followed by Bonferroni test for the comparison of multiple groups, and the significance was set at *P* < 0.05. The statistical graphs were prepared using GraphPad Prism Software (version 7).

## 3. Results

### 3.1. ADAM17 is Upregulated in Fibrosis Heart Tissues and MCFs Treated with TGF-*β*1

**Figure 1:**
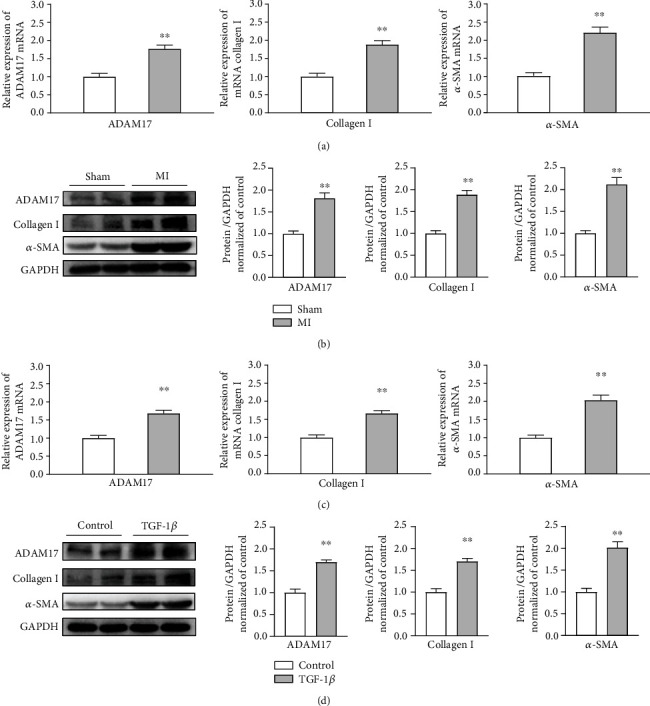
ADAM17 is upregulated in fibrosis heart tissues and mCFs treated with TGF-*β*1. Mice were divided into two groups: sham and MI. (a, b) The mRNA and protein levels of ADAM17, collagen I, and *α*-SMA in fibrosis heart tissues (*n* = 4). (c, d) mCFs were divided into two groups: control and TGF-*β*1 treatment (5 ng/mL, 48 h). The mRNA and protein levels of ADAM17, collagen I, and *α*-SMA in the process of mCF activation (*n* = 5). Data in (a–d) are expressed as mean ± SEM. ∗ indicates *P* < 0.05 and ∗∗ indicates *P* < 0.01 vs. sham or control groups.

To determine the potential mechanistic link between ADAM17 and cardiac fibrosis *in vivo*, a murine model of MI was established using a previously described protocol. The expression levels of fibrotic proteins, such as collagen I and *α*-SMA, were increased in the MI group. Meanwhile, ADAM17 mRNA and protein levels were significantly higher in fibrosis hearts than in normal hearts (Figures 1(a) and 1(b)). I*n vitro*, mCFs isolated from neonatal mice hearts were incubated with TGF-*β*1 (5 ng/mL, for 48 h). The mRNA and protein levels of collagen I and *α*-SMA were increased, indicating that the mCFs were effectively differentiated into myofibroblasts. Consistent with our results in the fibrosis heart tissues, the ADAM17 expression levels were higher in TGF-*β*1-treated mCFs than in those not subjected to TGF-*β*1 stimulation (Figures 1(c) and 1(d)). Taken together, these data indicate that ADAM17 may be involved in the regulation of cardiac fibrosis and activation of mCFs.

### 3.2. ADAM17 Knockdown Attenuated TGF-*β*1-Induced MCF Activation and the Upregulation of ECM Proteins

**Figure 2:**
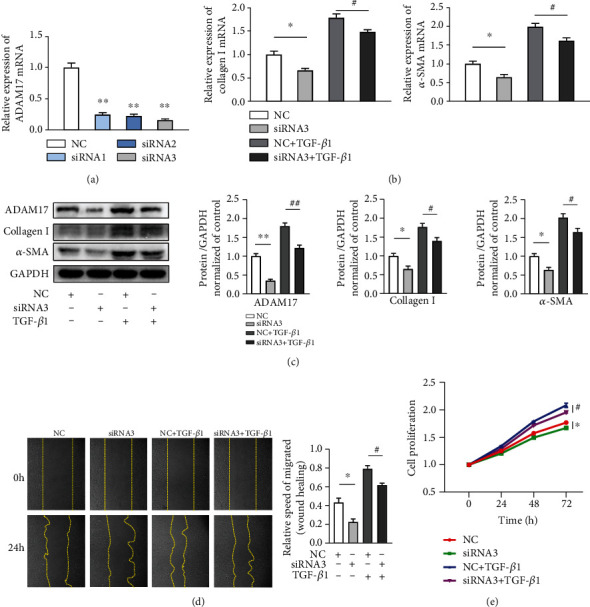
ADAM17 knockdown attenuated TGF-*β*1-induced mCF activation and the upregulation of ECM proteins. (a) The mRNA expression of ADAM17 after three siRNA chain transfections (*n* = 3); siRNA3 was the most efficient chain. (b, c) NC or siRNA3 transfection in the presence or absence of TGF-*β*1 (5 ng/mL, 48 h). The mRNA and protein levels of ADAM17, collagen I, and *α*-SMA in activated mCFs after transfection with NC or siRNA3 (*n* = 5). (d) The migration of mCFs (determined by the areas of cells protruding from the wound border) after transduction with NC or siRNA3 (*n* = 3). (e) The proliferation of mCFs was assayed using CCK-8 (*n* = 5). Data in (a–e) are expressed as mean ± SEM. ∗ indicates *P* < 0.05, and ∗∗ indicates *P* < 0.01 vs. the NC group; # indicates *P* < 0.05, and ## indicates *P* < 0.01 vs. the NC+TGF-*β*1 group.

To further identify the important role of ADAM17 in activation of mCFs, the cells were transfected with ADAM17-specific siRNA or scramble siRNA as a negative control (NC) for 24 h before treatment; qRT-PCR was used to confirm the knockdown efficiency of ADAM17. We found that transfection with siRNA3 reduced the ADAM17 levels by nearly 80%, compared to those in the NC groups (Figure 2(a)). Following the TGF-*β*1-induced activation of mCFs, the transcript and protein levels of the ECM protein collagen I, as well as those of the myofibroblast marker *α*-SMA, were increased. Further, siRNA3 successfully inhibited the expression of ADAM17, *α*-SMA, and collagen I (Figures 2(b) and 2(c)). Our data revealed that knockdown of ADAM17 not only inhibited mCF differentiation but also reduced the synthesis and secretion of collagen I. Next, we assessed the mCF migration and proliferation abilities using wound healing and CCK-8 assays, respectively. We found that TGF-*β*1 significantly increased the proliferation and migration abilities of mCFs; the knockdown of ADAM17 partially reversed these phenomena (Figures 2(d) and 2(e)). Thus, our results suggested that ADAM17 enhances the collagen synthesis and secretion, migration, and proliferation abilities of mCFs; that is, it promotes the activation of mCFs.

### 3.3. ADAM17 Regulates ER Stress and Mitophagy to Promote the Activation of MCFs

**Figure 3:**
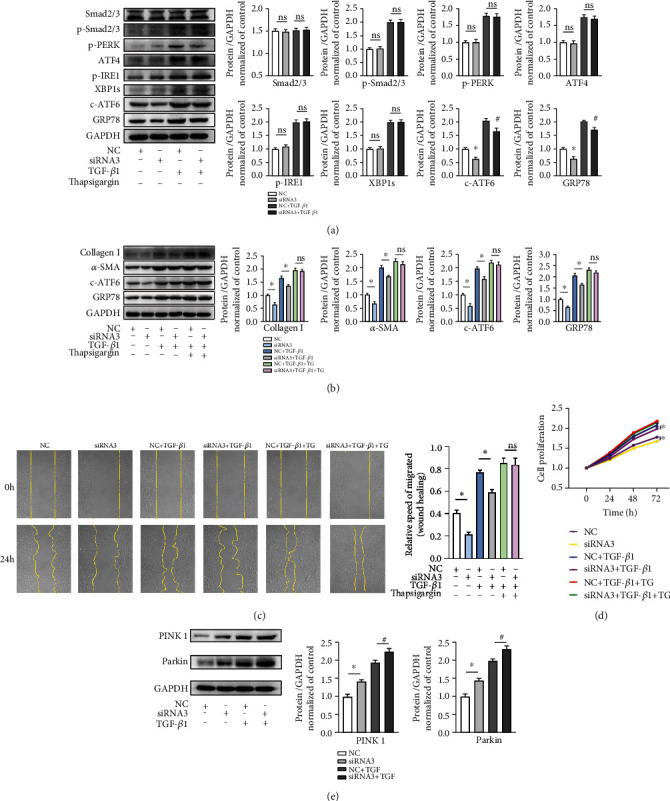
ADAM17 regulates ER stress and mitophagy to promote the activation of mCFs. (a) Protein expressions of Smad2/3, p-Smad2/3, p-PERK, ATF4, p-IRE1, XBP1s, c-ATF6, and GRP78 in mCFs with NC or siRNA3 transfection in the presence or absence of TGF-*β*1 (*n* = 5). (b) The protein levels of the ER stress branch, the ATF6 and GRP78, after transfection of NC or siRNA3 in the presence or absence of TGF-*β*1 or TG (*n* = 5). (c, d) Wound healing and CCK-8 assays for migration and proliferation in mCFs transfected with NC or siRNA3 in the presence or absence of TGF-*β*1 or TG (*n* = 5). (e) Protein expression of PINK1 and Parkin with NC or siRNA3 transfection in the presence or absence of TGF-*β*1 in mCFs (*n* = 5). Data in (a–e) are expressed as mean ± SEM. ns: not statistically significant; ∗ indicates *P* < 0.05, and ∗∗ indicates *P* < 0.01 vs. the NC or NC+TGF-*β*1 groups.

It is well-known that TGF-*β*1 signal through the activation of Smad2/3 (canonical pathway) to promote mCF differentiation, migration, and proliferation and the deposition of ECM proteins during the formation of pathological cardiac fibrosis [35, 36]. Therefore, we investigated the effects of ADAM17 on the TGF-*β*1/Smad2/3 pathway in mCFs. Western blotting analysis revealed that the phosphorylation levels of Smad2/3 were increased significantly after stimulation with TGF-*β*1, but they were unaltered between the NC and ADAM17 knockdown groups. These data indicated that ADAM17 had no effect on the canonical Smad2/3 pathway. In addition, TGF-*β*1 is known to trigger an increase in ER stress and the unfolded protein response. After the TGF-*β*1-induced activation of mCFs, the downstream signaling molecules associated with ER stress, phosphorylated PERK, and IRE1 (p-PERK and p-IRE1), ATF4, XBP1s, ATF6, and GRP78 were markedly upregulated. Then, we found that the activity of the ATF6 was further reduced after the silencing of ADAM17, compared to the case in the NC groups (Figure 3(a)). These results indicate that the ATF6, but not the PERK/ATF4 and IRE1/XBP1 pathways associated with ER stress, may mediate ADAM17-induced activation of mCFs.

To further investigate whether the ATF6 is involved in the activation of mCFs, we employ thapsigargin (TG), a key stimulator of acute ER stress. Compared with those in the TGF-*β*1 treatment groups, the administration of the TGF-*β*1 and TG groups not only increased the expression levels of ATF6 and GRP78, as expected, but also upregulated the expression levels of collagen I and *α*-SMA. However, after adding TG, there was no significant difference in the expression levels of collagen I, *α*-SMA, ATF6, and GRP78 between the siRNA3 group and the NC group (Figure 3(b)). Next, migration and proliferation abilities of mCFs also showed similar changes, according to the results of the wound healing and CCK-8 assays, respectively. Knockdown of ADAM17 inhibited mCF migration and proliferation abilities, but this difference disappeared after using TG (Figures 3(c) and 3(d)). Collectively, these data suggested that the decreased expression of ADAM17 protects against mCF activation via the inhibition of the ATF6 in ER stress.

A recent study reported that ADAM17 and mitophagy are involved in the occurrence and development of tumors [31]. To clarify whether mitophagy is involved in the regulation of mCF activation by ADAM17, we observed the mitophagy response in the process of mCF activation. PINK1/Parkin pathway acts as a classical regulation pathway in mitophagy and plays an important role in cardiac fibrosis [26, 37]. We found that the protein levels of PINK1 and Parkin were enhanced after TGF-*β*1 stimulation, and their protein levels were further increased after the knocking down ADAM17 (Figure 3(e)). These data showed that ADAM17 may regulate mCF activation by affecting mitophagy.

### 3.4. Inhibition of ADAM17 with TMI-005 Reduced the Degree of Post-MI Fibrosis and Enhanced Cardiac Function *in Vivo*

**Figure 4:**
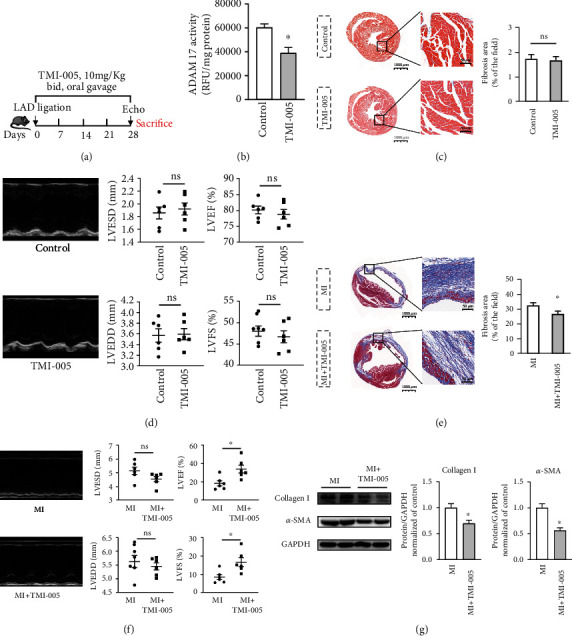
Inhibition of ADAM17 with TMI-005 reduced the degree of post-MI fibrosis and enhanced cardiac function *in vivo*. C57BL/6 mice were randomly divided into 4 groups, including control, TMI-005, MI, and MI treated with TMI-005. MI groups had left anterior descending ligation for 28 days to induced cardiac fibrosis. (a) Schema of the TMI-005 treatment protocol. (b) ADAM 17 activity in heart tissues was determined by measuring cleavage of the internally quenched fluorogenic substrate MCA-KPLGLDpa-AR-NH2 (*n* = 6). (c, d) In the control and TMI-005 groups, Masson's trichrome staining and M-mode images were used to assess the collagen deposition and cardiac function. LVESD, LVEDD, LVEF, and LVFS were quantified via echocardiography. (e, f) In the MI and MI treated with TMI-005 groups, Masson's trichrome staining and M-mode images were used to assess the degree of fibrosis and cardiac function. LVESD, LVEDD, LVEF, and LVFS were quantified via echocardiography. (g) Collagen I and *α*-SMA expression levels were quantified using western blotting. *n* = 6 in each group. Data in (a–g) are expressed as mean ± SEM. ∗ indicates *P* < 0.05, and ∗∗ indicates *P* < 0.01 vs. the control or MI groups.

To assess whether ADAM17 mediates post-MI cardiac fibrosis *in vivo*, we employed TMI-005, a pharmacological selective inhibitor of ADAM17. The 8-week-old mice were subjected to MI via permanent LAD ligation and then administered 10 mg/kg TMI-005 by oral gavage twice daily for 4 weeks, as depicted in Figure 4(a). We performed echocardiography and Masson's trichrome staining analyses to assess collagen deposition and cardiac function, respectively. After TMI-005 treatment alone for 4 weeks, ADAM17 activity was remarkably inhibited in the heart, and there was no significant difference in collagen deposition and cardiac function, compared to those in the control group (Figures 4(b)–4(d)). Then, we observed the heart function and degree of fibrosis in the mice post-MI after they were treated with TMI-005 for 28 days. The Masson's trichrome staining results showed that the ADAM17 inhibitor prevented MI-associated increase in the area of cardiac fibrosis (Figure 4(e)). In Figure 4(f), compared with the MI group, although the TMI-005 administration group had no obvious difference in LVESD and LVEDD, the LVEF and LVFS were improved. And the western blotting results also showed that the levels of the fibrotic proteins collagen I and *α*-SMA were reduced in the hearts of the mice treated with the ADAM17 inhibitor (Figure 4(g)). Thus, the ADAM17 inhibitor TMI-005 notably improved cardiac function after MI. These findings support the speculation that ADAM17 inhibition exerts protective effects on fibrosis post-MI and cardiac function.

## 4. Discussion

Despite the rising prevalence and the adverse prognosis associated with cardiac fibrosis in both men and women, there is still no effective treatment for this condition. Although ADAM17 exerts potential regulatory effects in cardiovascular diseases, including cardiac hypertrophy, coronary microvascular dysfunction, and thoracic aortic aneurysm [12, 13, 15, 38–40], its precise function in cardiac fibrosis remains unknown. In this study, we demonstrate the key role of ADAM17 in mCF activation and heart fibrosis progression, which can provide a new direction for antifibrosis therapies. We first found that ADAM17 was upregulated in RNA and protein levels in both fibrosis heart tissue and activated mCFs. To verify the role of ADAM17 in fibrosis, we transfected mCFs with specific siRNA to knock down the expression of ADAM17; we found that silencing of ADAM17 in the mCFs further prevented the deposition of ECM proteins and abrogated the TGF-*β*1-induced increase in the proliferation and migration abilities *in vitro*. Consistent with existing studies in other systems, increased expression of ADAM17 can promote lung and kidney fibrosis [41, 42]. Therefore, we speculate that the upregulated expression of ADAM17 in cardiac fibrosis is harmful to mCFs.

ADAM17 is well known as a membrane-bound enzyme that can regulate growth and development by mediating the catalytic shedding of various growth factors and cytokines. However, an immunohistochemical study found that most of the active form of ADAM17 were also localized in the cellular perinuclear region, with a small amount present in the plasma membrane [11]. Whether the ADAM17 in the cytoplasm affects the activation of mCFs is still unclear. The major finding of the present study is that the ATF6 branch of ER stress contributes to the development of ADAM17-related cardiac fibrosis. As we know, *in vitro* and *in vivo* evidences have shown that the TGF-*β*1-induced increase in ECM protein synthesis occurs mainly through the canonical Smad2/3 pathway [35, 36]. The present study showed that the downregulation of ADAM17 via siRNAs further alleviated mCF activation via the ATF6 branch, but not the PERK/ATF4, IRE1/XBP1s, and canonical Smad2/3 pathway. Interestingly, a recent study also found that vascular ADAM17 is indirectly related to ER stress with regard to regulating Ang II-induced cardiovascular remodeling [15]. Based on the observations of the present study, we suggest that the downregulation of ADAM17 attenuates the degree of mCF activation by inhibiting the ATF6 branch of ER stress. However, the specific mechanism between ATF6 and ADAM17 is still unclear and needs further study.

Mitophagy is essential for the clearance of dysfunctional mitochondria to maintain the mitochondrial integrity; dysregulation of mitophagy is associated with many cardiovascular diseases [26]; the absence of mitophagy leads to the aggravation of cardiac fibrosis [43]. It is regulated through multiple pathways, and the most well studied in the cardiovascular system is the canonical PINK1/Parkin pathway. Enhanced Pink/Parkin-mediated mitophagy can decrease the degree of fibrosis, as well as improved cardiac function, and stabilize the microvascular network [30]. Other reports demonstrate that the PINK1/Parkin pathway involved in heart repair following MI [37]. Our study showed that ADAM17 affects mitophagy to regulate the activation of mCFs. Consistent with previous studies, we found that the knockdown of ADAM17 reduced the degree of mCF activation by further activating the PINK1/Parkin pathway. However, the specific mechanism between mitophagy and ADAM17 is still unclear and needs further study.

Numerous studies have shown that there is a physical and functional interaction between the ER and mitochondria, and this interaction is involved in the regulation of cardiovascular diseases. Excessive ER stress will aggravate abnormal Ca^2+^ and ROS regulation, further damage mitochondria, exacerbate dysregulation of mitophagy, lead to cell death, and eventually promote cardiac fibrosis [21–23]. Bueno et al. found that in aging lung epithelial cells, PINK1 can be activated by ER stress, which can damage mitochondrial function and aggravate pulmonary fibrosis [44]. When Zhang et al. studied brain tissue ischemia-reperfusion injury, they also found that moderate ER stress can activate mitophagy through ATF4 to protect the tissues [45]. In a study of pulmonary arterial hypertension, Dromparis et al. found that ATF6 produced by ER stress in pulmonary artery smooth muscle cells can improve mitochondrial function by reducing abnormal ER and mitochondrial Ca^2+^ transfer and inhibiting key calcium-sensitive mitochondrial enzymes [23]. All the above studies suggest that there is a complex relationship between ER stress and mitophagy. This study is the first to indicate that ADAM17, ER stress, and mitophagy play an important role in the regulation of cardiac fibrosis, but the specific mechanism between them requires further study.

Since ADAM17 was discovered in 1997, many inhibitors have been developed that work through the following mechanisms: (1) inhibition of ADAM17 expression and induction, (2) inhibition of ADAM17 maturation, (3) inhibition of ADAM17 activation, (4) inhibition at the active site by small molecules, (5) inhibitory prodomains, and (6) inhibition of substrate recognition. Among the above inhibitors, most of them were developed in the early stage, had poor targeting, and were only used in vitro, while some of them used in the mouse had been proved to damage in other organs [46–48]. Recently, a phase II trial with TMI-5 for treatment of rheumatoid arthritis showed no toxicity of this compound [49]. Therefore, we chose TMI-005 as the inhibitor *in vivo*. In this study, we used a cardiac fibrosis model established via LAD ligation for 28 days; decreased heart function and collagen deposition are common features of this model. We administered TMI-005 to the mice by oral gavage daily to inhibit ADAM17 in their heart tissues. Continuous oral ADAM17 inhibitors can markedly inhibit the activity of ADAM17 and do not affect the collagen deposition and cardiac function in normal hearts. In cardiac fibrosis models, inhibiting ADAM17 expression can alleviate cardiac fibrosis and improve cardiac function. This phenomenon may be due to the fact that ADAM17 is not the main molecule regulating ECM deposition and cardiac function under a physiological state. Collectively, our *in vivo* and *in vitro* experiments revealed that the deficiency of ADAM17 inhibited the activation of mCFs, protected against cardiac fibrosis, and improved heart function.

## 5. Conclusion

Our indirect evidence indicates that ADAM17 plays a key role in cardiac fibrosis. Knockdown of ADAM17 inhibits the activation of cardiac fibroblasts by regulating the ATF6 branch of ER stress and the PINK1/Parkin pathway of mitophagy. Inhibition of ADAM17 is associated with beneficial effects of against post-MI cardiac fibrosis and improving heart function. These data provide insights into novel mechanisms with potential treatment strategies for cardiac fibrosis.

## Data Availability

The data used to support the findings of this study are available from the corresponding author upon request.

## Supplementary Materials

Supplementary Materials Table s1: sequences of siRNAs. Table s2: sequences of primers.
