# Supplementary material for: The Downregulation of ADAM17 Exerts Protective Effects against Cardiac Fibrosis by Regulating Endoplasmic Reticulum Stress and Mitophagy
Source: Oxid Med Cell Longev. 2021 May 6;2021:5572088. doi: 10.1155/2021/5572088 (PMC8118735; doi:10.1155/2021/5572088)
Supplement: Supplementary Materials — Table s1: sequences of siRNAs. Table s2: sequences of primers. [file 5572088.f1.docx]

**Supplementary Materials**

Table s1.Sequences of siRNAs

| si-m-ADAM17_001 | GGACCAAGGAGGAAAGTAT |
| --- | --- |
| si-m-ADAM17_002 | CTACAAGACCATAGAAAGT |
| si-m-ADAM17_003 | GGTATGGAGTGCAGATAGA |

Table s2. Sequences of primers

| Genes | Sequence of Primers (forward/reverse) |
| --- | --- |
| Collagen I | 5′-CTTCACCTACAGCACCCTTGTG-3′ |
|  | 5′-CTTGGTGGTTTTGTATTCGATGAC-3′ |
| α-SMA | 5′-CCCAGACATCAGGGAGTAATGG-3′ |
|  | 5′-TCTATCGGATACTTCAGCGTCA-3′ |
| ADAM17 | 5′-AGGACGTAATTGAGCGATTTTGG-3′ |
|  | 5′-TGTTATCTGCCAGAAACTTCCC-3′ |
| GAPDH | 5′-GGTGGACCTCATGGCCTACA-3′ |
|  | 5′-CTCTCTTGCTCAGTGTCCTTGCT-3′ |
